# Effects of purple sweet potato polysaccharide on performance, egg quality characteristics, jejunal morphology, and gut microbiota of Hy-Line Brown laying hens

**DOI:** 10.1016/j.psj.2024.104366

**Published:** 2024-09-27

**Authors:** Cecilia T. Oluwabiyi, Zhigang Song

**Affiliations:** Department of Animal Science and Technology, Key Laboratory of Efficient Utilization of Non-grain Feed Resources, Shandong Agricultural University, Tai'an City, Shandong Province 271018, China

**Keywords:** 16S rRNA, cecal microbiota, intestinal morphology, laying hen, purple sweet potato polysaccharide

## Abstract

This study was conducted to investigate the effects of dietary purple sweet potato polysaccharide (**PSPP**) supplementation on production performance, egg quality characteristics, jejunal morphology, and gut microbiota modulation of Hy-line Brown laying hens. A total of 288 23-wk-old Hy-line Brown laying hens were randomly divided into 1 of the 4 dietary treatment groups, with 6 replicates and 12 laying hens per replicate. The 4 groups were fed basal diet supplemented with varying concentrations of PSPP (0, 1, 2, or 4 g/kg) for 6 wks. At the end of the feeding trials, eggs were collected for egg quality analysis, jejunal samples were collected for morphology assessment, and cecal content was analyzed by 16S rRNA high-throughput sequencing to determine intestinal microbiota. Experimental treatments did not differ regarding laying performance and egg quality. However, polynomial contrast analysis showed that there was a linear decrease (*P* = 0.042) in yolk color. The jejunal morphology did not differ among the treatment groups. The alpha and beta diversity were not different between the treatment groups. The cecal microbiota was dominated by *Bacteroidota* and *Firmicutes* at the phylum level and Bacteroides at the genus level. The relative abundance of *Firmicutes* was increased (*P* = 0.012) and *Bacteroidota* was decreased (*P* = 0.009) in the cecal content of PSPP2. *Firmicutes* to *Bacteroidota* ratio increased (*P* = 0.005) in the PSPP2 group. Polynomial contrast analysis showed that PSPP had a quadratic effect on *Firmicutes* to *Bacteroidota* ratio (*P* = 0.004) and on the relative abundance of *Firmicutes* (*P* = 0.006) and *Bacteroidota* (*P* = 0.006). At the genus level, increasing PSPP level showed a pattern of linear increase (*P* = 0.046) in *[Ruminococcus]_torques_group* and linear decrease (*P* = 0.015) in the *Rikenellaceae_RC9_gut_group*. It can be concluded that PSPP altered the gut microbiota, but did not influence jejunal morphology or laying performance and egg quality of laying hens. Further research is recommended to fully understand the potential and determine the optimal level of PSPP in laying hens.

## INTRODUCTION

The gut microbiota and its metabolites play crucial roles in maintaining host health in poultry. Antibiotics have been widely used to optimize the gut microbiota and enhance the health and productivity of poultry. However, antibiotics have led to the development of antimicrobial drug resistance and may cause dysbiosis (Abreu et al., 2023; Tran Nguyen Minh et al., 2023). Dysbiosis is linked to the development of intestinal diseases in laying hens (Pourabedin and Zhao, 2015; Beldowska et al., 2023). In addition, antibiotic residues in poultry products have been an issue of serious concern to consumers (Muaz et al., 2018), and as a result, many countries have banned the use of antibiotics in poultry production. Therefore, the use of natural feed additives has emerged and received concerted efforts in recent years as safer alternatives to antibiotics to enhance health and productivity in poultry. Among others, polysaccharides extracted from different sources have been used in different forms in animal production for their numerous health benefits (Guo et al., 2022; Van Doan et al., 2023; Yang et al., 2023).

Polysaccharides have been proven to possess prebiotic, immunomodulatory, anti-inflammatory, and antioxidant properties without having side effects (Li et al., 2010; Sun et al., 2018; Murphy et al., 2023). Studies have shown that polysaccharides enhance intestinal health via gut microbiota modulation (Zhang et al., 2022a,c). Polysaccharides can bypass digestion to a large extent in humans and animals and reach the hindgut, where they can be fermented by gut microbes; thus, the beneficial effect of polysaccharides is microbiota-dependent (Gan et al., 2022). The fermentation of polysaccharides therefore increases the abundance of beneficial bacteria and inhibits the proliferation of pathogenic ones (Choi et al., 2015; Wu et al., 2023).

Purple sweet potato, belonging to the family Convolvulaceae, is a member of the sweet potato family. As an edible root vegetable, it has become a popular healthy food due to its high nutritional value (Mohanraj and Sivasankar, 2014; Alam, 2021). Purple sweet potato contains various bioactive components, such as polysaccharides, anthocyanins, vitamins, amino acids, and minerals (Sun et al., 2018; Yong et al., 2019; Alam, 2021; Tang et al., 2023). Purple sweet potato polysaccharide (**PSPP**) is mainly composed of d-rhamnose, d-arabinose, d-galactose, d-glucose, d-xylose, d-mannose, and glucuronic acid (Bie et al., 2021). Several studies have reported the beneficial effect of PSPP on the regulation of the gut microbiota of mice (Tang et al., 2018; Bie et al., 2021). However, there is a scarce report on the effect of PSPP on laying hens. This study hypothesized that supplementation of PSPP in laying hens' diet may have beneficial effects on performance and intestinal health by exerting modulatory effects on the gut microbiota. Hence, the present study aimed to study the efficacy of supplementing graded concentrations of PSPP on laying performance, egg quality, jejunal morphology, and gut microbiota in laying hens.

## MATERIALS AND METHODS

### Animals and Experimental Design

A total of two hundred and eighty-eight, 23-wk-old Hy-line Brown laying hens with similar laying performance were randomly divided into 4 treatment groups with 6 replicates of 12 laying hens each: PSPP0, PSPP1, PSPP2, and PSPP4, with the birds fed a basal diet (16.2% CP, 2700 kcal/kg) supplemented with PSPP at 0, 1, 2, or 4 g/kg of diet, respectively. The dosage was based on a previous study, in which 1 or 2.5 g/kg polysaccharide was recommended for improved production performance in laying hens (Guo et al., 2020). The experimental basal diet composition is presented in Table 1. The same batch of PSPP purchased from Huaxing Biotechnology Co., Ltd. (Xi'an, China) was used throughout this study, and according to the manufacturer, the polysaccharide content in PSPP was 50%. A 7-wk feeding experiment was conducted, with the first week for acclimatization, and the birds were fed the experimental diet, which was in mash form for the subsequent 6 wk. The birds were housed in 2-tier battery cages, with 3 chickens per cage, and hens in 4 adjacent cages were considered a replicate. The birds were reared in a well-ventilated room with a 16L: 8D light schedule and were not exposed to stress.

**Table 1:** Composition and nutrient levels of basal diet (air-dry basis, %).

Ingredients	Content, %	Nutrient level^3^	Content
Corn, 8.5% CP	61.00	Crude protein, %	16.2
Soybean meal, 43% CP	24.76	Metabolizable energy, kcal/kg	2700
Wheat bran	2.00	Calcium, %	3.50
Soybean oil	1.30	Nonphytate P, %	0.35
Limestone	9.01	NaCl, %	0.35
CaHPO_4_	1.15	Lysine, %	0.761
NaCl	0.33	Methionine, %	0.362
DL-Met, 98%	0.10	Lys + Met, %	0.650
Choline Chloride, 50%	0.10	Threonine, %	0.657
Vitamin premix^1^	0.05	Tryptophan, %	0.215
Mineral premix^2^	0.20		
Total	100.00		

1 Vitamin premix provided the following per kg of diets: vitamin A (retinyl acetate), 8 000 IU; vitamin D3 (cholecalciferol), 1 600 IU; vitamin E (DL-α-tocopheryl acetate), 5.0 IU; vitamin K, 0.5 mg; vitamin B1 0.8 mg, vitamin B2, 2.5 mg; vitamin B6, 3.0 mg; vitamin B12, 0.004 mg; niacin, 20.0 mg; pantothenic acid (calcium pantothenate), 2.2 mg; biotin, 0.10 mg; folic acid, 0.25 mg.

2 Mineral premix provided the following per kg of diets: Mn, 60 mg; Zn, 80 mg; Fe, 60 mg; I, 0.35 mg; Cu, 8.0 mg; Se, 0.30 mg.

3 Nutrient levels are calculated values.

### Production Performance and Egg Quality

During the experiment, the eggs laid were picked, counted, and weighed daily, while the feed consumption was recorded weekly. The data recorded were used to calculate the egg production, egg weight, feed intake, and feed conversion ratio (**FCR**) based on the different periods (1–3 wk, 4–6 wk, and 1–6 wk). Average egg weight was calculated by dividing the total egg weight by the total number of eggs. The FCR was defined as the ratio of average feed intake to egg mass. At the end of the feeding trial, thirty eggs (5 eggs per replicate) were randomly collected from each treatment for egg quality analysis. The length and width were measured using a digital vernier caliper, and the shape index was calculated as the ratio of the length to the width. The shell color and thickness were measured at 3 locations (equator, blunt, and sharp ends), and the values recorded at the 3 locations were averaged. The shell color was measured using a color difference reader (CR-10 Plus, Konica Minolta Inc., Japan), while the shell thickness was measured using an eggshell thickness gauge (Robotmation Co., Ltd., Japan). Then, shell strength was measured using an Egg Force Reader (ORKA Food Technology, Israel). The egg weight, Haugh unit, albumen height, and yolk color were measured using an Egg Analyzer (ORKA Food Technology, Israel). The yolk was separated from the albumen using an egg separator and weighed.

### Sample Collection

At the end of the feeding trial, 1 hen per replicate was randomly selected for analysis. The hens were manually slaughtered (Yang et al., 2024), and the liver and spleen were excised, weighed, and expressed as a percentage of the live body weight. The mid-jejunum samples were collected and fixed in formalin for morphological analysis. The cecal contents were collected into sterile tubes, quickly frozen in liquid nitrogen, and stored at -80°C.

### Jejunal Morphology Analysis

The formalin-fixed jejunum samples were dehydrated, paraffin-embedded, stained with hematoxylin and eosin (**H&E**), and observed under a light microscope for the measurement of the villus height, crypt depth, and villus/crypt ratio. Three intact villi per bird were selected for measurement.

### 16S rRNA-Based Microbiota Analysis

Total genomic DNA was extracted from the cecal content of laying hens using cetyltrimethylammonium bromide, and the purity and concentration of the extracted DNA were determined using 1% agarose gel electrophoresis. Based on the concentration, DNA was diluted with sterile water to a concentration of 1 ng/μl, and the V3–V4 region of the bacteria 16S rRNA gene was amplified by PCR. The resulting PCR products were detected with electrophoresis on 2% agarose gel and purified using the Qiagen Gel Extraction Kit (Qiagen, Germany) according to the manufacturer's protocol.

The TruSeq® DNA PCR-Free Sample Preparation Kit (Illumina, USA) was used to generate the sequencing libraries following the manufacturer's instructions, and the library quality was evaluated on the Qubit Fluorometer (Thermo Scientific). Then, sequencing and bioinformatics were performed on an Illumina NovaSeq platform by Novogene Bioinformatics Technology Co. Ltd. (Tianjin, China).

Raw tags were filtered to obtain clean and quality tags, chimera sequences were removed, and sequences with 97% similarity were clustered into operational taxonomic units (**OTU**) by QIIME2. The alpha diversity indices (Chao1, Shannon, and Simpson) and beta diversity of the cecal microbiota were calculated with QIIME2 and displayed with R software. Beta diversity was determined using the Bray-Curtis index and visualized as principal coordinate analysis (**PCoA**) and nonmetric multidimensional scaling (**NMDS**) plots.

### Statistical Analysis

Statistical analyses were performed using SPSS 29.0 software (IBM Inc., Chicago, IL). Replicate was considered an experimental unit for performance data, n = 6 for each group. Five eggs from each replicate were considered as an experimental unit for egg quality traits, n = 30. A laying hen was considered an experimental unit for organ index, jejunal morphology, and microbiota analysis data, n = 6. Data were checked for normality with the Shapiro-Wilk test. Normally distributed data were analyzed based on 1-way ANOVA and the differences between means were compared using Tukey's post hoc test. Non-normal data were analyzed with the Kruskal–Wallis test. Data are presented as means with SEM. Linear or quadratic effects were evaluated by orthogonal polynomial contrasts. Differences were considered significant when *P* < 0.05.

## RESULTS

### Production Performance and Egg Quality

Production performance data are presented in Table 2. The dietary inclusion of PSPP did not influence the production performance of laying hens, including feed intake, egg production, egg weight, and feed conversion ratio, during the different stages of the experiment (1–3 wk and 4–6 wk) and the entire feeding trial from 1 to 6 wk. Table 3 shows the effect of dietary inclusion of PSPP on egg quality. The dietary PSPP had a linear effect (*P* = 0.042) and tended to decrease the yolk color. However, there were no differences in the other egg quality characteristics among the treatment groups.

**Table 2:** Effects of purple sweet potato polysaccharide (**PSPP**) on production performance in laying hens.

Items	Dietary PSPP level, g/kg				*P-*value			
	0	1	2	4	SEM	PSPP	Linear	Quadratic
1–3wk								
Feed intake, g	115	113	114	112	0.62	0.251	0.101	0.948
Egg production, %	92.7	90.0	90.7	88.6	1.12	0.374	0.285	0.888
Average egg weight, g	56.7	56.8	56.2	56.4	0.18	0.566	0.329	0.936
Daily egg mass, g	52.6	51.1	51.0	50.0	0.66	0.429	0.216	0.869
Feed conversion ratio	2.19	2.22	2.24	2.24	0.02	0.944	0.564	0.862
4–6wk								
Feed intake, g	119	119	120	118	0.66	0.819	0.893	0.509
Egg production, %	94.0	92.7	92.7	89.4	1.12	0.740	0.201	0.671
Average egg weight, g	58.3	58.9	58.1	58.1	0.16	0.341	0.434	0.383
Daily egg mass, g	54.8	54.6	53.9	52.0	0.66	0.474	0.154	0.541
Feed conversion ratio	2.18	2.19	2.24	2.29	0.02	0.543	0.167	0.747
1–6wk								
Feed intake, g	117	116	117	115	0.57	0.399	0.346	0.673
Egg production, %	93.3	91.3	91.7	89.0	1.01	0.540	0.190	0.874
Average egg weight, g	57.5	57.9	57.2	57.3	0.16	0.466	0.358	0.680
Daily egg mass, g	53.7	52.9	52.4	51.0	0.58	0.461	0.128	0.800
Feed conversion ratio	2.19	2.20	2.24	2.26	0.26	0.740	0.278	0.936

Data were presented as mean with SEM (n = 6).

**Table 3:** Effects of purple sweet potato polysaccharide (**PSPP**) on egg quality of laying hens.

Items	Dietary PSPP level, g/kg					*P*-value		
	0	1	2	4	SEM	PSPP	Linear	Quadratic
Egg weight, g	50.4	50.8	51.6	50.1	0.33	0.520	0.951	0.169
Egg shape index	1.26	1.26	1.25	1.27	0.003	0.441	0.492	0.204
Eggshell color, L*	54.9	54.4	54.8	55.2	0.29	0.851	0.599	0.527
Eggshell color, a*	20.8	21.2	21.3	20.6	0.13	0.559	0.768	0.042
Eggshell color, b*	29.3	29.4	29.2	28.7	0.17	0.658	0.239	0.429
Eggshell thickness, mm	0.37	0.36	0.35	0.36	0.002	0.335	0.244	0.206
Eggshell strength, N	46.8	45.5	47.8	47.6	0.65	0.611	0.439	0.703
Haugh unit	78.1	80.0	78.0	82.6	1.29	0.229	0.319	0.598
Albumen height, mm	6.00	6.27	6.05	6.53	0.15	0.352	0.311	0.735
Yolk color	5.73	5.33	5.53	5.13	0.08	0.079	0.042	1.000
Yolk weight, %	28.5	28.4	28.3	28.9	0.19	0.715	0.518	0.367

Data were presented as mean with SEM (n = 30).

L* = lightness; a* = redness; b* = yellowness.

### Jejunal Morphology, and Organ Index

As shown in Table 4, there were no differences in jejunal villus height, crypt depth, and villus-to-crypt ratio among the treatment groups. The liver, and spleen index of laying hens were similar among the treatment groups.

**Table 4:** Effect of purple sweet potato polysaccharide (PSPP) on jejunal morphology and organ index of laying hens.

Items	Dietary PSPP level, g/kg					*P-*value		
	0	1	2	4	SEM	PSPP	Linear	Quadratic
Villus height, µm	1213	1150	1170	1164	39.5	0.922	0.744	0.739
Crypt depth, µm	295	269	292	254	16.9	0.824	0.553	0.863
VH: CD	4.33	4.45	4.15	4.92	0.21	0.641	0.456	0.466
Liver index, %	1.52	1.69	1.86	1.69	0.05	0.228	0.181	0.138
Spleen index, %	0.12	0.15	0.13	0.17	0.01	0.198	0.089	0.756

VH: CD, villus height: crypt depth.

Data were presented as mean with SEM (n = 6).

### Summary of Sequence Analysis

**Figure 1:**
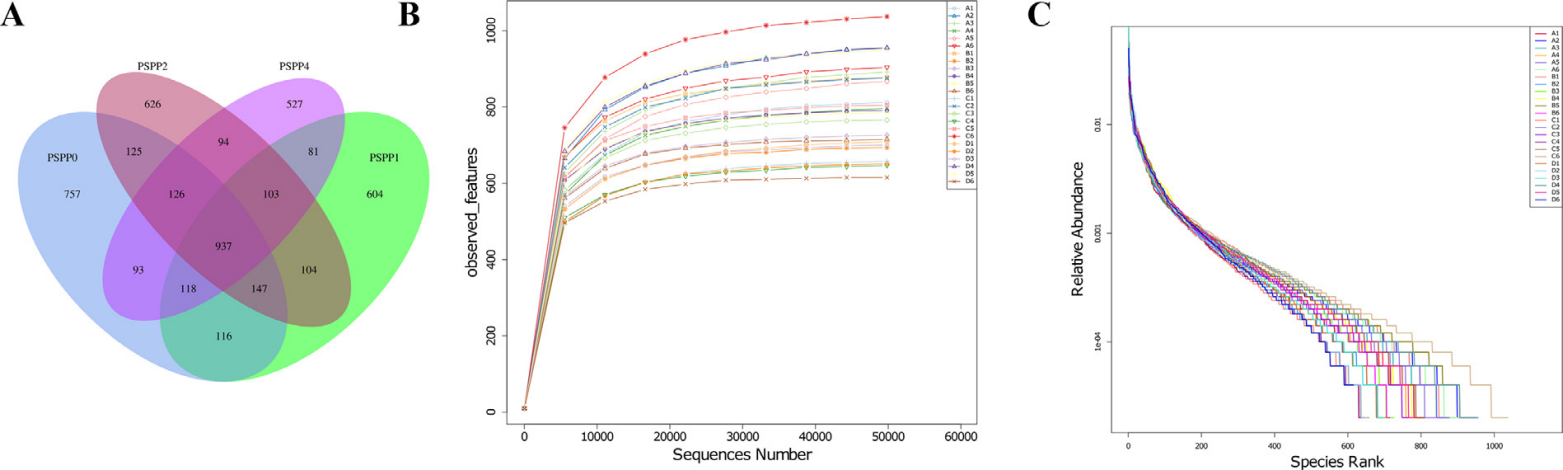
Summary of sequence data. (A)Venn diagram for the OTUs of cecal microbiota of laying hens fed with PSPP; (B) Alpha rarefaction of the observed features of cecal microbiota of laying hens fed with PSPP; (C) Rank abundance curves of the cecal microbiota of laying hens fed with PSPP. PSPP. PSPP0, PSPP1, PSPP2, and PSPP4 represented the data from laying hens fed with 0, 1, 2, and 4 g PSPP per kilogram of the diet, respectively. A1-A6 = PSPP0; B1-B6 = PSPP1; C1-C6 = PSPP2; D1-D6 = PSPP4.

A total of 937 OTUs were shared among the 4 dietary groups, while 757, 604, 626, and 527 OTUs were unique to PSPP0, PSPP1, PSPP2, and PSPP4 (Figure 1A), respectively. The rarefaction curve illustrating the depth of the sequencing is shown in Figure 1B. The samples analyzed reached a plateau, as shown in the rarefaction curves, indicating that the depth of the sequencing was sufficient and covered almost all the taxa. The rank abundance curves for all samples were wide and fell gently, indicating satisfactory evenness and abundance (Figure 1C).

### Diversity of Cecal Microbiota of laying hens

**Figure 2:**
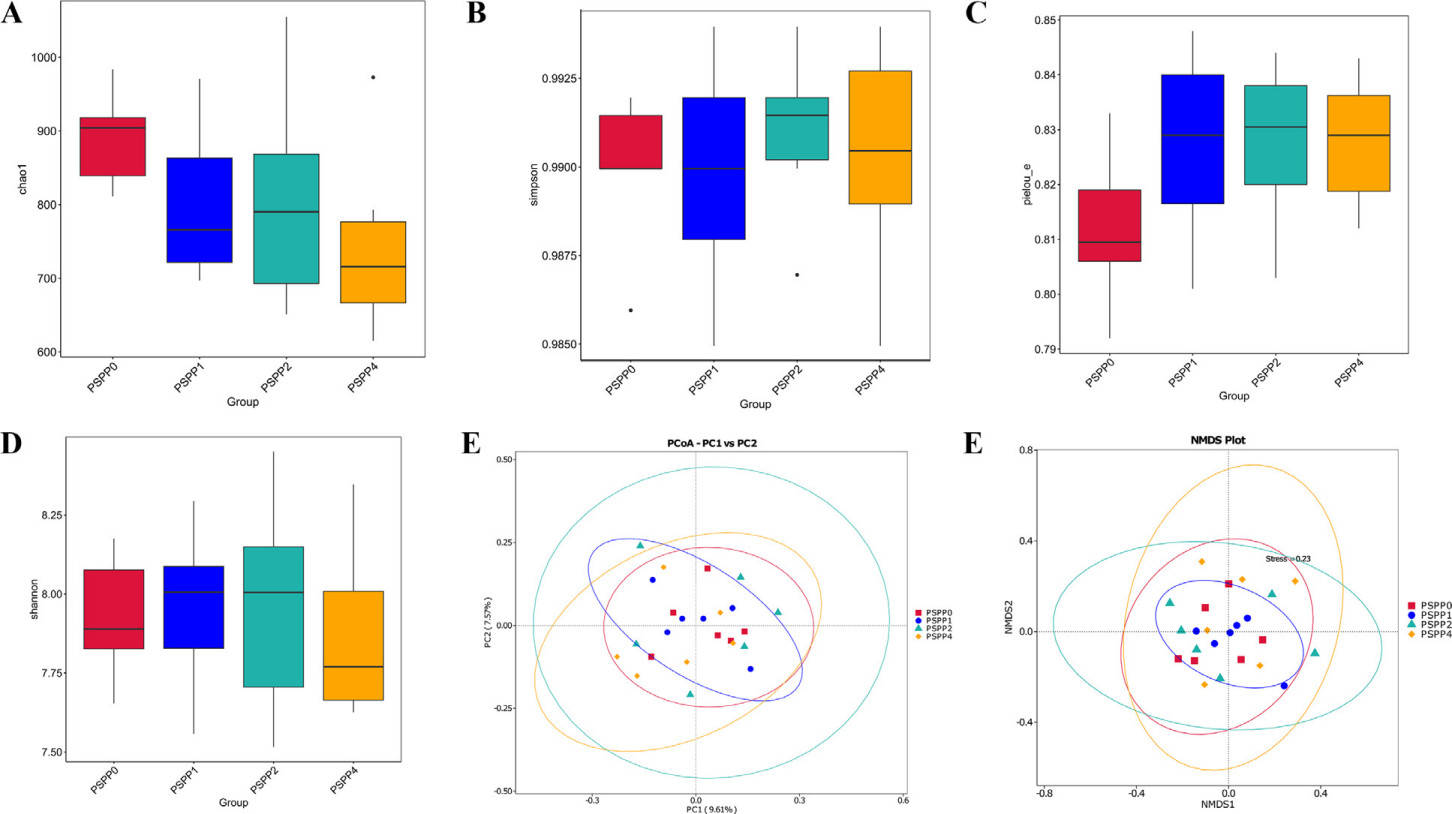
Alpha and beta diversity assessed by (A) Chao, (B) Simpson, (C) Pielou, and (D) Shannon, (E) Principal coordinate analysis (PCoA), and (F) Nonmetric multidimensional scaling (NMDS) in the cecal contents of laying hens fed diets supplemented with varying levels of PSPP. PSPP0, PSPP1, PSPP2, and PSPP4 represented the data from laying hens fed with 0, 1, 2, and 4 g PSPP per kilogram of the diet, respectively.

The alpha diversity indices, including chao1 (Figure 2A), Simpson (Figure 2B), Pielou (Figure 2C), and Shannon (Figure 2D), were not different among the treatment groups. The cecal microbiota in the treatment groups were presented in 4 intersecting clusters, presented as PCoA (Figure 2E) and NMDS (Figure 2F) plots, indicating that dietary supplementation with PSPP did not influence the beta diversity of gut microbiota.

### Relative Abundance of the Cecal Microbiota at the Phylum Level

**Figure 3:**
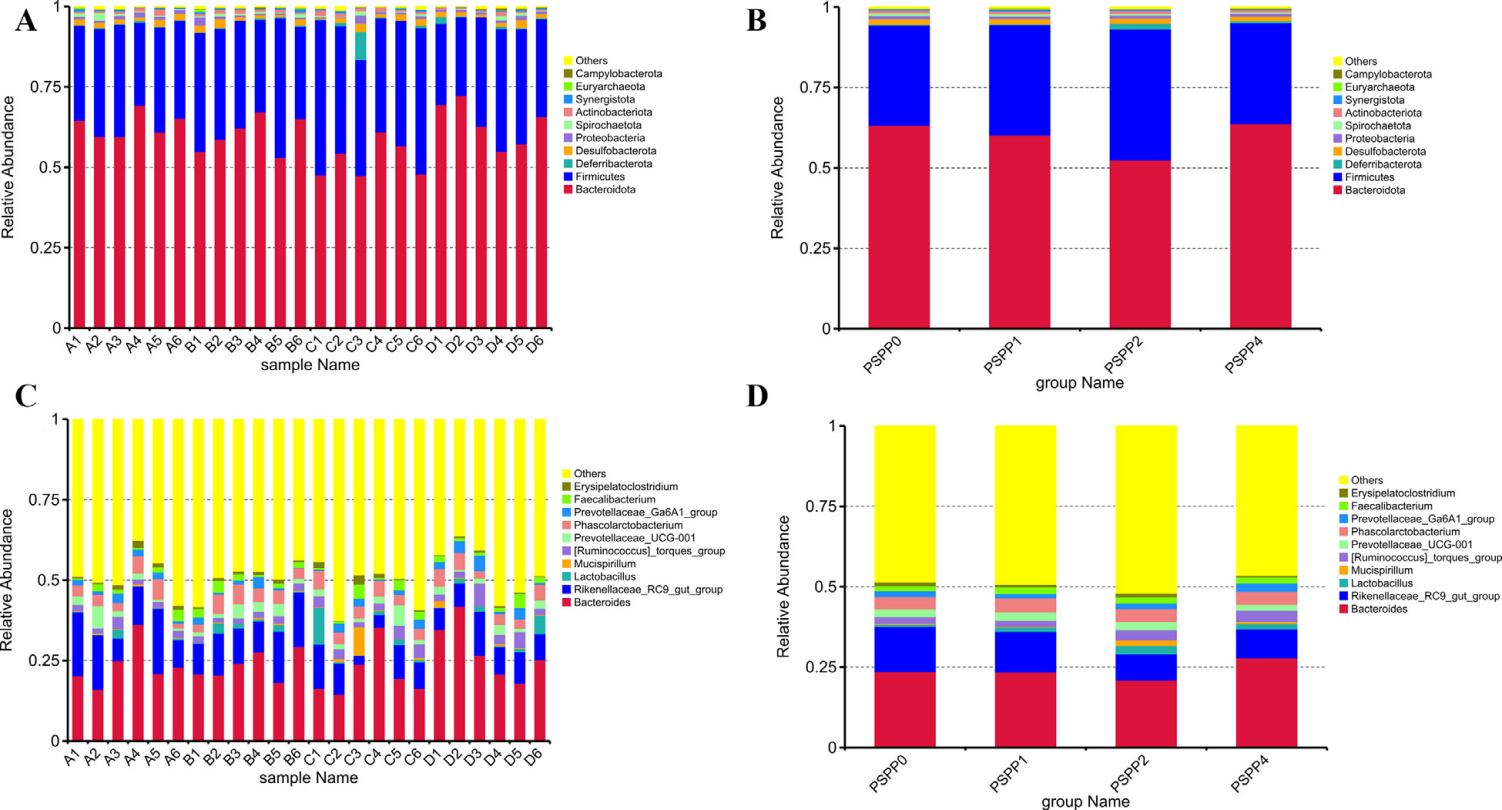
Effects of purple sweet potato polysaccharide on the relative abundances of top 10 cecal microbiota. (A, B) Phylum level; (C, D) genus level. PSPP0, PSPP1, PSPP2, and PSPP4 represented the data from laying hens fed with 0, 1, 2, and 4 g PSPP per kilogram of the diet, respectively.

The bacterial community analysis showed that the top 10 phyla were similar in all 4 treatment groups, as illustrated in Figure 3A and B, but with differences in their relative abundance. *Bacteroidota* (52.51 to 63.76%), and *Firmicutes* (31.16 to 40.63%) were the dominant microbiota in the 4 dietary treatments, followed by *Desulfobacterota* (1.50 to 1.69%), *Proteobacterota* (0.82 to 1.06%), *Deferribacterota* (0.25 to 1.81%), *Actinobacteriota* (0.59 to 0.99%), *Spirochaetota* (0.48 to 0.83%), *Synergistota* (0.30 to 0.56%), *Euryarchaeota* (0.01 to 0.19%), and *Campylobacterota* (0.04 to 0.27%). The phyla of the cecal microbes with a relative abundance greater than 1% in each group are shown in Table 5. Compared to the control group, the PSPP2 group increased the abundance of *Firmicutes* (*P* = 0.012) and reduced the relative abundance of the phylum *Bacteroidota* (*P* = 0.009). In addition, the *Firmicutes to Bacteroidota ratio* was higher in the PSPP 2 group compared with the CON group (*P* = 0.005). Dietary PSPP supplementation had quadratic effects on *Firmicutes to Bacteroidota ratio* (*P* = 0.004), and the relative abundance of *Firmicutes* (*P* = 0.006) and *Bacteriodota* (*P* = 0.006). However, no difference was found in the relative abundance of the other phyla among the treatment groups.

**Table 5:** Effect of purple sweet potato polysaccharide (**PSPP**) on relative abundance of Bacteria at the Phylum level.

Items	Dietary PSPP level, g/kg					*P-*value		
	0	1	2	4	SEM	PSPP	Linear	Quadratic
*Firmucutes/Bacteroidota*	0.49^b^	0.58^ab^	0.78^a^	0.50^b^	0.03	0.005	0.380	0.004
*Bacteroidota, %*	63.1 a	60.2^ab^	52.5^b^	63.7 a	1.41	0.009	0.569	0.006
*Firmicutes, %*	31.1 b	34.2^ab^	40.6^a^	31.3^b^	1.24	0.012	0.463	0.006
*Desulfobacterota, %*	1.69	1.63	1.60	1.49	0.13	0.965	0.621	0.920

Data were presented as mean with SEM (n = 6).

a,b Means in the same row are different P < 0.05.

### Relative Abundance of the Cecal Microbiota at the Genus Level

**Figure 4:**
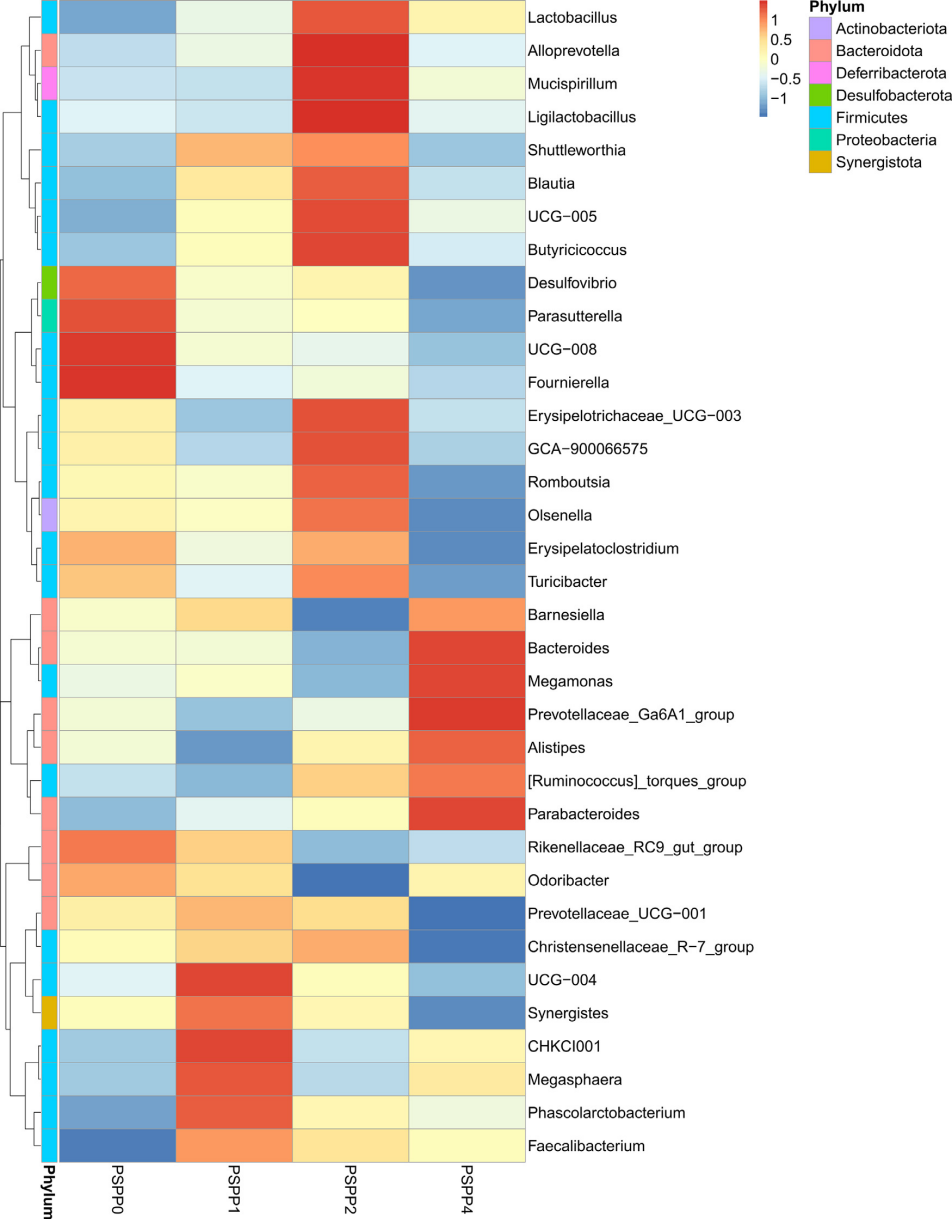
Heatmap of relative species abundance in the cecal content of laying hens at the genus level. PSPP0, PSPP1, PSPP2, and PSPP4 represented the data from laying hens fed with 0, 1, 2, and 4 g PSPP per kilogram of the diet, respectively.

The bacterial community analysis showed that the top 10 genera were present in all the 4 treatment groups as illustrated in Figures 3C and D. At the genus level, the predominant taxa were *Bacteroides* (20.98 to 27.85%), *Rikenellaceae*_RC9_gut_group (8.13–14.11%), *Lactobacillus* (0.63–2.62%), *Mucispirillum* (0.25–1.81%), *Ruminococcus*_torques_group (1.92–3.47%), *Prevotellaceae*_UCG-001 (1.89–2.56%), *Phascolarctobacterium* (3.78–4.38%), *Prevotellaceae*_Ga6A1_group (1.39–2.59%), *Faecalibacterium* (1.65–2.04%), and *Erysipelatoclostridium* (0.42–1.14%). The heatmap provides a visual representation of the relative species abundance in the cecal content of laying hens at the genus level (Figure 4), and the genera of the cecal microbes with a relative abundance greater than 1% in each group are shown in Table 6. Dietary PSPP supplementation had linear effects on *[Ruminococcus]_torques_group* (*P* = 0.046) and *Rikenellaceae_RC9_gut_group* (*P* = 0.015)*,* and feeding 2 g/kg of PSPP tended to decrease the *Rikenellaceae_RC9_gut_group* population. However, no difference was found in the relative abundance of the other genus among the treatment groups.

**Table 6:** Effect of purple sweet potato polysaccharide (**PSPP**) on relative abundance of bacteria at the genus level.

Items	Dietary PSPP level, g/kg					*P-*value		
	0	1	2	4	SEM	PSPP	Linear	Quadratic
*Bacteroides, %*	23.5	23.4	20.9	27.8	1.46	0.342	0.438	0.248
*Phascolarctobacterium, %*	3.77	4.38	4.10	4.00	0.27	0.909	0.881	0.557
*[Ruminococcus]_torques_group, %*	2.17	1.92	3.09	3.47	0.28	0.226	0.046	0.563
*Faecalibacterium, %*	1.65	2.04	1.95	1.89	0.25	0.825	0.789	0.683
*Rikenellaceae_RC9_gut_group, %*	14.1	12.6	8.1	9.00	0.93	0.056	0.015	0.493
*Prevotellaceae_Ga6A1_group, %*	1.77	1.39	1.71	2.59	0.25	0.425	0.248	0.235
*Prevotellaceae_UCG-001, %*	2.41	2.55	2.48	1.88	0.33	0.901	0.609	0.609

Data were presented as mean with SEM (n = 6).

### LEfSe Analysis

**Figure 5:**
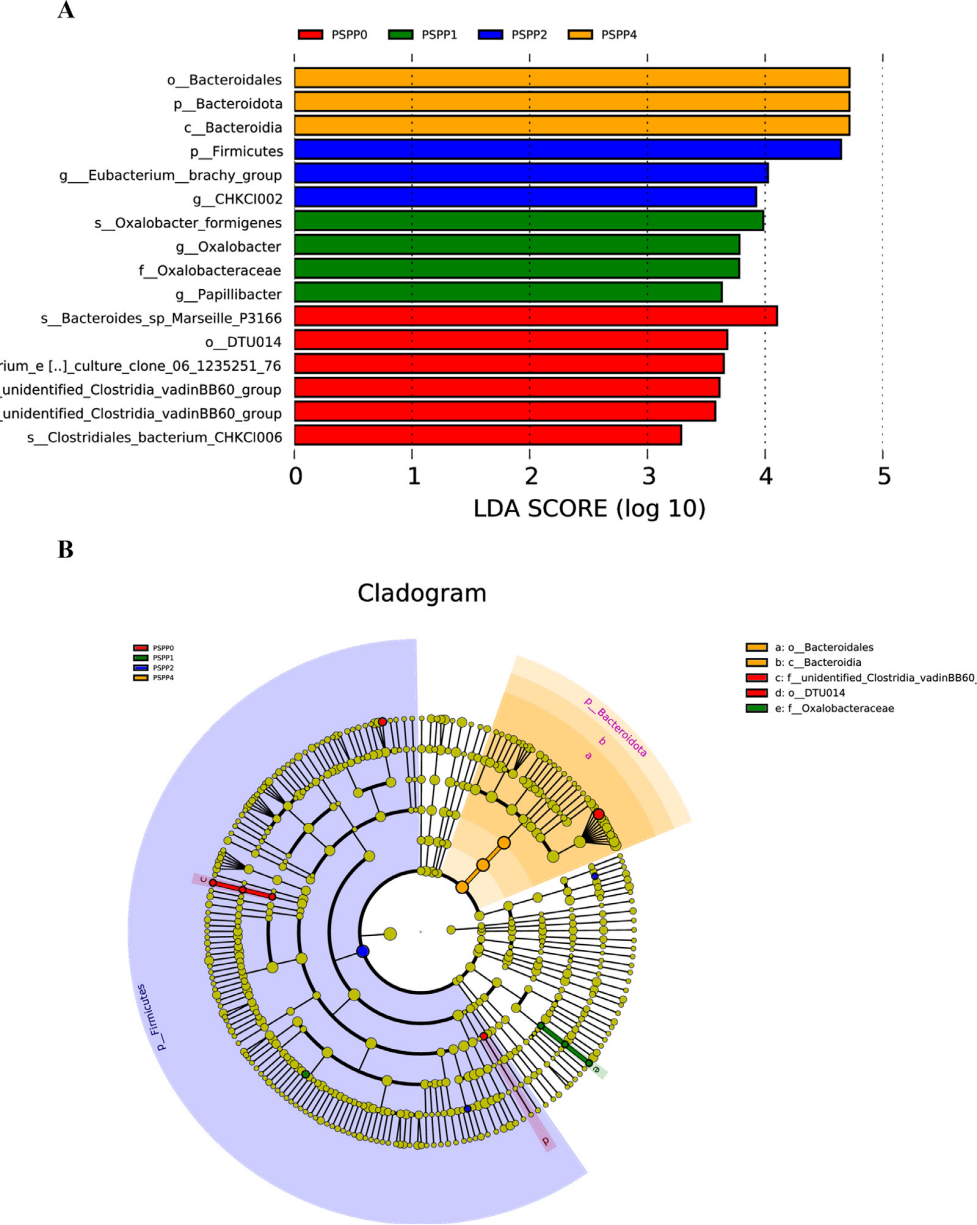
(A) An LDA score histogram showing microbes in the cecal contents of laying hens by Linear discriminant analysis (LDA) effect size method (LEfSe) with LDA score > 2. (B) Cladogram showing the phylogenetic distribution of gut microbiota in cecal contents of laying hens. PSPP0, PSPP1, PSPP2, and PSPP4 represented the data from laying hens fed with 0, 1, 2, and 4 g PSPP per kilogram of the diet, respectively.

The differential microbes in the cecal contents of laying hens identified by LEfSe analysis with LDA score > 2 is shown in Figure 5A, and the cladogram generated by LEfSe analysis is shown in Figure 5B. In PSPP0, s_*Bacteroides* sp. *Marseille-P3166*, o_DTU014, s_*Clostridiales* bacterium enrichment culture clone 06-1,235,251-76, *unidentified_Clostridia_vadinBB60_group*, and s_*Clostridiales* bacterium CHKCI006 markedly increased. In PSPP1(green) s_*Oxalobacter formigenes, g_Oxalobacter*, f_Oxalobacteraceae, and *g_Papllllbacter* markedly increased*.* In PSPP2 (blue), *p_Firmicutes, g_Eubacterium*__brachy_group and g_CHKCI002 markedly increased. In PSPP4 (orange). o_*Bacteroidales*, p_*Bacteroidota*, and c_*Bacteroidia* markedly increased.

## DISCUSSION

Polysaccharides from different sources have been supplemented in the diets of laying hens to enhance performance and immunity and modulate the gut microbiota (Yu et al., 2021; Zhou et al., 2023). In this study, dietary supplementation of 1, 2, or 4 g/kg PSPP did not influence the laying performance (feed intake, egg production, egg weight, and FCR) of laying hens. To the best of our knowledge, there is no previous report on the effect of PSPP on the production performance of laying hens. However, our findings corroborate those of Shi et al. (2024), who reported that dietary supplementation of *Radix isatidis* polysaccharide did not affect laying performance.

Enhancing egg quality through the manipulation of the laying hen diet has been a focus in poultry production (Wang et al., 2017). In this study, the effect of PSPP supplementation on egg quality characteristics was evaluated. Notably, both internal and external characteristics are important indicators of egg quality in poultry (Schwägele, 2011). Here, we observed that the dietary supplementation of PSPP did not influence the egg quality traits, including egg shape index, shell thickness, shell strength, albumen height, Haugh unit, and yolk index. Similar to the findings of this study, previous studies with dandelion polysaccharides (Cao et al., 2023) and Comfrey polysaccharides (Zhou et al., 2022) showed that polysaccharide supplementation did not affect the egg quality of laying hens. However, in an earlier study, Lv et al. (2021) reported an increase in yolk weight, yolk color, and eggshell thickness by supplementation with 400 mg/kg of astragalus polysaccharide in chongren hens. The discrepancy observed in the experimental results may be influenced by differences in the age, breed, and management of the hens.

Our study reveals that PSPP did not influence the spleen and liver index in laying hens. Similar to the findings of this experiment, Tang et al. (2018) fed PSPP to normal and cyclophosphamide-treated mice by oral gavage and observed that the immune organ index (thymus and spleen) of normal mice was not affected by the treatment. Furthermore, Chen et al. (2020) reported that mice administered *Pueraria lobata* polysaccharide had similar organ weights.

Intestinal morphology in terms of villus height and crypt depth has been one of the parameters that have been validated to measure the absorptive capacity of the intestine. Longer villi with shorter crypt depth indicate an increased surface area for nutrient absorption (Marchewka et al., 2021). In the present study, none of the dietary treatments influenced the jejunal morphology. Similar to our findings, Adhikari et al. (2018) reported that fructooligosaccharides did not influence ileal morphology in laying hens. However, contrary to this study, a previous study showed that *Radix isatidis* polysaccharide supplementation increased jejunal villus height and decreased the crypt depth of laying hens (Shi et al., 2024). The differences observed between the reported effects of polysaccharides on intestinal morphology might be due to the varying sources, supplemental levels, and rearing conditions across the studies.

Gut microbiota plays crucial roles in various metabolic and physiological processes in the body. It is well known that gut microbiota prevents leaky gut, and acts as a defense against pathogenic invasion through secreting antimicrobial peptides, and competing for nutrients and adhesion sites, thus playing an important role as barriers to enhancing intestinal health (Takiishi et al., 2017; Di Vincenzo et al., 2024). Homeostasis of the intestinal microbiota has been linked to overall health in both human and animal species, and several studies have shown that dysbiosis is the cause of many diseases (Afzaal et al., 2022). As natural feed additives, polysaccharides possess gut microbiota modulation properties, and studies have shown the beneficial effect of polysaccharides on the gut microbiota in different parts of the gut in poultry and other animal species (Yu et al., 2021; Cao et al., 2022; Xiao et al., 2022). In this study, we investigated the effects of dietary supplementation of PSPP on gut microbiota in Hy-line Brown laying hens.

Alpha and beta diversity are important and known indicators of species richness and evenness used in gut microbiota studies. In the present study, dietary supplementation of PSPP did not influence the α- diversity index such as Chao, Simpson, Shannon, and Pielou. In addition, beta diversity was not affected, indicating that the species richness and evenness were similar among the treatment groups. In agreement with the findings of this study, a previous study by Yu et al. (2021) has shown that dietary supplementation with enteromorpha polysaccharides did not influence the diversity of the ileal microbial community. This confirms the findings of previous research that, while species abundance can be affected by changes in diet or rearing environment, once the microbial population is established, the presence or absence of microbes is less susceptible to changes (Ovi et al., 2021; Zhang et al., 2022b).

In the current study, *Firmicutes* and *Bacteriodota* are the dominant phyla in the cecal content of laying hens, which is consistent with the findings of previous studies (Wei et al., 2013; Dong et al., 2017). Here, PSPP2 increased the relative abundance of *Firmicutes* in the cecal content of laying hens. Similar to the findings of the current study, Yu et al. (2021) reported an increase in the abundance of *Firmicutes* in the ileal contents of laying hens fed diet supplemented with 0.2% Enteromorpha polysaccharides. Furthermore, PSPP2 increased the *Firmicutes to Bacteroidota* ratio in the cecal contents of laying hens. An increased *Firmicutes to Bacteroidota* ratio can enhance energy absorption and metabolism, and improve animal performance (Chen et al., 2023). This suggests that 2 g/kg is the optimal dosage that could alter the relative abundance in the 2 dominant phyla.

Similar to previous studies, *Bacteroides, Rikenellaceae_RC9_gut_group*, and *[Ruminococcus]_torques_group* accounted for the largest proportion of the total cecal microbial community of laying hens at the genus level (Xu et al., 2023). Furthermore, Bacteroides is the dominant genus which is consistent with the results of previous studies (Zhang et al., 2022d; Chen et al., 2023). Although similar microbial richness and evenness were observed in the current study, distinct bacterial taxa were enriched in the treatment groups indicating that the dietary supplementation of PSPP influences the microbiota composition in the cecum of laying hens. However, the dosages of PSPP used in this study did not influence the performance or intestinal morphology of the laying hens, indicating that the minimal changes observed in the gut microbiota did not confer benefits to the host in these regards. Therefore, further research with varying levels of PSPP is warranted.

In conclusion, purple sweet potato polysaccharide altered the cecal microbiota but neither influenced intestinal morphology nor the production performance and egg quality in laying hens. Further studies is warranted to fully understand the potential and determine the optimal level of PSPP in laying hens.

## DISCLOSURES

The authors declare no conflict of interest
